# A Comparison of Depression and Mental Distress Indicators, Rhode Island Behavioral Risk Factor Surveillance System, 2006

**Published:** 2011-02-15

**Authors:** Yongwen Jiang, Jana Earl Hesser

**Affiliations:** Center for Health Data and Analysis, Rhode Island Department of Health. Dr Jiang is also affiliated with the Department of Community Health, The Warren Alpert Medical School of Brown University, Providence, Rhode Island; Center for Health Data and Analysis, Rhode Island Department of Health, Providence, Rhode Island, and Department of Community Health, The Warren Alpert Medical School of Brown University, Providence, Rhode Island

## Abstract

**Introduction:**

Depression is a public health concern that warrants accurate population estimates. The patient health questionnaire 8 (PHQ-8) offers high sensitivity and specificity for assessing depression but is time-consuming to administer, answer, and score. We sought to determine whether 1 of 3 simpler instruments — the shorter PHQ-2 or 2 single questions from the health-related quality of life (HRQOL) module of the Behavioral Risk Factor Surveillance System (BRFSS) — could offer accuracy comparable to the PHQ-8.

**Methods:**

We compared the depression and mental distress indicators of 2006 Rhode Island BRFSS data by using 4 types of analyses: 1) sensitivity and specificity estimates, 2) prevalence estimates, 3) multivariable logistic regression modeling of the relationship between each of the 4 indicators and 11 demographic and health risk variables, and 4) geographic distribution of prevalence.

**Results:**

Compared with the PHQ-8, the 3 other measures have high levels of specificity but lower sensitivity. Depression prevalence estimates ranged from 8.6% to 10.3%. The adjusted odds ratios from logistic regression modeling were consistent. Each of the indicators was significantly associated with low income, being unable to work, current smoking, and having a disability.

**Conclusion:**

The PHQ-8 indicator is the most sensitive and specific and can assess depression severity. The HRQOL and PHQ-2 indicators are adequate to obtain population prevalence estimates if questionnaire length is limited.

## Introduction

Depression is a public health problem. In 2000, it was rated as the fourth leading cause of disease globally, accounting for 4.4% of total disability adjusted life years (1-3). Depression affects approximately 14.8 million American adults, or 6.7% of the population (4), and is linked to risk behaviors such as smoking, alcohol use, and physical inactivity (5-7). It is also associated with chronic diseases such as diabetes, asthma, arthritis, cardiovascular disorders, and cancer (2,8). Depression is underdiagnosed and undertreated (1,2,6). Getting a reliable estimate of depression prevalence in the general population is important but presents challenges. Several measures exist for detecting depression in clinical settings (9), and multiple self-reported questions used for clinical diagnosis have been adapted for use on population-based surveys (6,10). The challenge is finding a simple means to estimate depression prevalence in the general population.

The Behavioral Risk Factor Surveillance System (BRFSS) is a telephone survey administered in all 50 states, the District of Columbia, Puerto Rico, the US Virgin Islands, and Guam with funding and specifications from the Centers for Disease Control and Prevention (CDC) (11). The BRFSS monitors the prevalence of behavioral risks for the leading causes of disease and death among adults in the United States (11). A 9-question health-related quality of life (HRQOL) module has been available since 1995 (12). In 2006, a 10-question depression and anxiety (D&A) module was also made available to states. Rhode Island was the only state to include both modules on its 2006 BRFSS questionnaire.

We compared depression and mental distress estimates from the HRQOL and D&A modules using data from Rhode Island's 2006 BRFSS. Two of the 5 items that are related to mental health in the HRQOL module were used, 1 for *sad/blue/depressed* and 1 for *frequent mental distress*. Two measures from the D&A module were used. The D&A module includes the patient health questionnaire 8 (PHQ-8), which is used to create a 5-point scale for depression severity based on an algorithm using responses to 8 questions. Severity scores can be grouped to create a dichotomous variable for *current depression* (6,9). The first 2 questions of the PHQ-8, called the PHQ-2, is also used to provide a simple measure of *current depression* (9). Our hypothesis was that either of the HRQOL questions or the PHQ-2 can serve as a proxy for the PHQ-8 on the BRFSS. The objective of this study was to assess whether 1 or 2 questions on depression and mental distress can yield prevalence estimates of depression comparable to those from the PHQ-8, which has a high degree of sensitivity and specificity.

## Methods

### Study design

We used the 2006 Rhode Island BRFSS for this analysis, as this was the only year to include both the HRQOL and the D&A modules. From January through December 2006, the Rhode Island BRFSS conducted random-digit–dialed telephone interviews with 4,515 Rhode Island adults aged 18 or older. A detailed description of BRFSS methods, including survey design, random sampling, and weighting procedures, is available from the CDC BRFSS website (11). Information about Rhode Island's 2006 BRFSS is available from the Rhode Island Department of Health's website (13).

### Depression and mental distress indicators

Two depression and mental distress questions are on the HRQOL module. They asked respondents to estimate how many days in the past 30 days they experienced the following: "felt sad, blue, or depressed" (14 or more days = frequent depressive symptoms), and "mental health, which includes stress, depression, and problems with emotions, was not good" (14 or more days = frequent mental distress). The authors selected the 14-day minimum period because clinicians and clinical researchers often use this period as a marker for clinical depression disorders. In addition, most of the publications we reviewed that use the BRFSS HRQOL indicators use the cutoff of 14 or more days (14-23). Adopting this precedent ensured comparability with other studies.

The PHQ-8 contains 8 of the 9 criteria for diagnosis of major depression as defined in the fourth edition of the *Diagnostic and Statistical Manual of Mental Disorders*. These questions ask the respondent to indicate how many days each of the following has occurred in the past 2 weeks: 1) had little interest or pleasure in doing things; 2) felt down, depressed, or hopeless; 3) had trouble falling asleep or staying asleep or sleeping too much; 4) felt tired or had little energy; 5) had a poor appetite or ate too much; 6) felt bad about yourself, or felt that you were a failure or had let yourself or your family down; 7) had trouble concentrating on things, such as reading the newspaper or watching television; 8) moved or spoke so slowly that other people could have noticed, or being so fidgety or restless that you were moving around a lot more than usual. The number of days for each question is converted to points (0-1 day = 0 points; 2-6 days = 1 point; 7-11 days = 2 points; and 12-14 days = 3 points), and the number of points is totaled for the 8 questions to determine a depressive symptoms severity score (6,9). If a response to any of the 8 questions was missing, a score was not calculated. Five severity categories are defined: no, mild, moderate, moderately severe, and severe depression. For the dichotomous variable, a score of 0 to 9 points, which is no and mild depression, was defined as no depression, while a score of 10 to 24 points, which was the other 3 categories, was defined as current depression (6,9,24). The PHQ-2 is the first 2 questions of the PHQ-8 that inquire about depressed mood and anhedonia. A score of 0 to 2 points is defined as no depression; a score of 3 to 6 points is defined as current depression (9). If a response to either of the 2 questions was missing, a score was not calculated. The proportion of records with missing values for the 2 single HRQOL items was 1.3% and 1.4%; for the PHQ-2, 7.9%; and for the PHQ-8, 11.4%.

### Risk factors, health conditions, and demographics

For the analysis, we chose 3 health risk behaviors: current smoking, chronic alcohol use, and no leisure-time physical activity (PA); 4 health conditions: asthma, diabetes, obesity, and physical disability; and 4 demographic measures: age, sex, income, and employment status. We selected these risk and demographic factors based on our earlier work (25). We dichotomized some covariates for the analysis (ie, sex, current smoking, alcohol use, PA, asthma, diabetes, obesity, and disability), and the other covariates had multiple categories (ie, age, income, and employment status). The definitions of the 11 covariates are available in our previous article (25).

### Analysis

Multiple imputation has been extensively applied to account for missing data in survey samples (26,27). To maintain maximal sample size and retain all valid data, we simulated missing data for all variables using multiple imputation. In our study, depending on the analytical model, 24% to 30% of the 4,515 records in our data set had missing data for 1 or more of the 11 predictor or 4 outcome variables. Therefore, to retain all records, we imputed missing values for age, sex, race, marital status, education, employment status, health insurance, smoking, drinking, PA, asthma, diabetes, obesity, disability, square-root transformed income, the 5 mental health items in the HRQOL module, and the 10 D&A items. Analyzing the data without imputation did not change our conclusions (25).

Results for these different indicators were compared in 4 ways. First, using the PHQ-8 indicator as the standard, we compared the sensitivity and specificity of the 3 simpler measures. Second, we compared prevalence estimates generated by the 4 measures. Third, using multivariable logistic regression, we compared the relationship between each of the indicators and 11 demographic and health risk variables. Finally, we compared the geographic distribution of prevalence for 2 of the 4 indicators using geographic information system (GIS) mapping.

SAS version 9.1 (SAS Institute, Inc, Cary, North Carolina) was used for all analyses because it can adjust for the BRFSS complex sampling design. We calculated the sensitivity and specificity of the 2 HRQOL indicators and the PHQ-2 indicator, compared with the PHQ-8. Four logistic regression models were used to calculate adjusted odds ratios (AORs) and 95% confidence intervals (CIs) to assess the effect of each of the 11 risk factors for each of the depression indicators. All statistical inferences were based on a significance level of *P* (2-sided) < .05 calculated by using the Wald *χ* ^2^ test. The results of analyses for each of the indicators were compared with one another.

ArcGIS 9.0 (Environmental Systems Research Institute, Inc, Redlands, California) was used to map depression prevalence estimates by cities and towns by zip code. We chose to use the Jenks Optimization (also called Jenks Natural Breaks Classification method) to create the value ranges of sad/blue/depressed and PHQ-8 current depression depicted on the GIS maps.

We chose to use natural breaks rather than defined interval classification as used by others (28,29). Defined interval classification allowed us to specify an interval by which to equally divide a range of attribute values. We wanted to judge whether the distributions depicted in the GIS graphs are consistent with one another. With defined interval classification, similar features can be placed in adjacent classes, or features with widely different values can be put in the same class. The resulting maps can be misleading.

## Results

Compared with the PHQ-8, each of the 3 indicators had a high level of specificity, ranging from 94.4% to 96.4%; the PHQ-2 had a slightly higher negative predictive value than the other 2 indicators (Table 1). The sensitivity of the 3 indicators was weaker, ranging from 59.4% to 66.8%. The PHQ-2 had higher sensitivity than HRQOL indicators. The positive predictive values for the PHQ-2 and the sad/blue/depressed indicator were almost identical; the "frequent mental distress" indicator had a lower positive predictive value.

In the HRQOL module, the prevalence of frequent mental distress among Rhode Island adults was 10.3% and of sad/blue/depressed was 8.9% (Table 2). In the PHQ-2 and PHQ-8, the prevalence of current depression was 9.8% and 8.6%, respectively. Results of tests for significance for sad/blue/depressed and for PHQ-8 were consistent for the 11 demographic and risk variables with the exception of results for age. Results of tests for significance for PHQ-2 and for PHQ-8 were consistent for the 11 demographic and risk variables with the exception of results for age and sex. Results of tests for significance for frequent mental distress and for PHQ-8 were consistent for all of the 11 demographic and risk variables. Prevalence estimates for the 11 demographic and risk variables for each of the indicators were comparable with one another.

We used the area under receiver operating curves (AUC) to assess model discrimination. The AUC for frequent mental distress, sad/blue/depressed, PHQ-2, and PHQ-8 were 0.74, 0.80, 0.78, and 0.83, respectively. The AORs for sad/blue/depressed and for PHQ-8 (Table 3) were consistent with one another for 6 predictors. Both indicate increased odds of depression for women, annual income less than $25,000, unable to work, current smoker, no leisure-time PA, and disability. The AORs for PHQ-2 and for PHQ-8 are consistent with one another for 6 predictors. Both indicate increased odds of depression for younger adults, annual income less than $25,000, unable to work, current smoker, no leisure time PA, and disability. The AORs for frequent mental distress and for PHQ-8 are consistent with one another for 8 predictors. Both indicate increased odds of depression for adults aged 18 to 44 years and 45 to 64 years, annual income less than $25,000, unable to work, current smoker, asthma, obesity, and disability.

**Figure 1:**
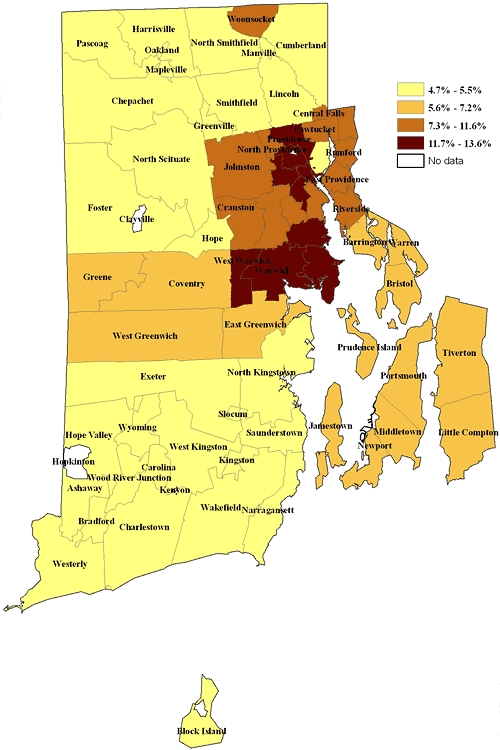
Prevalence of sad/blue/depressed, by Rhode Island cities and towns by zip code, data from the Health Related Quality of Life module of the 2006 Rhode Island Behavioral Risk Factor Surveillance System.

The prevalence of sad/blue/depressed in Providence (excluding the affluent east side), West Warwick, and Warwick ranged from 11.7% to 13.6%, higher than the rest of the state (Figure 1). The prevalence in Woonsocket, Central Falls, Pawtucket, North Providence, Johnston, Rumford, East Providence, Cranston, and Riverside ranged from 7.3% to 11.6%. These areas with higher depression rates include the more urban areas of the state, which have a higher proportion of low-income households than do the suburban and rural areas.

**Table:** 

**Town or City, Zip Code**	**Sad/Blue/Depressed, %**
Adamsville, 02801	6.88
Albion, 02802	5.50
Ashaway, 02804	4.74
Barrington, 02806	6.92
Block Island, 02807	4.74
Bradford, 02808	4.74
Bristol, 02809	6.92
Carolina, 02812	4.74
Central Falls, 02863	9.74
Charlestown, 02813	4.74
Chepachet, 02814	5.50
Coventry, 02816	7.22
Cranston, 02910	9.58
Cranston, 02920	9.58
Cranston, 02921	9.58
Cumberland, 02864	5.50
East Greenwich, 02818	7.22
East Providence, 02914	10.49
Exeter, 02822	4.74
Fiskeville, 02823	9.58
Forestdale, 02824	5.50
Foster, 02825	5.50
Glendale, 02826	5.50
Greene, 02827	7.22
Greenville, 02828	5.50
Harmony, 02829	5.50
Harrisville, 02830	5.50
Hope Valley, 02832	4.74
Hope, 02831	5.50
Jamestown, 02835	6.88
Johnston, 02919	11.55
Kingston, 02881	4.74
Lincoln, 02865	5.50
Little Compton, 02837	6.88
Manville, 02838	5.50
Mapleville, 02839	5.50
Middletown, 02842	6.41
Narragansett, 02882	4.74
Newport, 02840	6.41
North Kingstown, 02852	4.74
North Kingstown, 02854	4.74
North Providence, 02911	11.55
North Scituate, 02857	5.50
North Smithfield, 02896	5.50
Oakland, 02858	5.50
Pascoag, 02859	5.50
Pawtucket, 02860	9.74
Pawtucket, 02861	9.74
Pawtucket, 02862	9.74
Portsmouth, 02871	6.88
Providence, 02901	13.60
Providence, 02902	13.60
Providence, 02903	13.60
Providence, 02904	13.60
Providence, 02905	9.58
Providence, 02906	5.34
Providence, 02907	13.60
Providence, 02908	13.60
Providence, 02909	13.60
Providence, 02912	13.60
Prudence Island, 02872	6.92
Riverside, 02915	10.49
Rockville, 02873	4.74
Rumford, 02916	10.49
Saunderstown, 02874	4.74
Slatersville, 02876	5.50
Slocum, 02877	4.74
Smithfield, 02917	5.50
Tiverton, 02878	6.88
Wakefield, 02879	4.74
Wakefield, 02880	4.74
Warren, 02885	6.92
Warwick, 02886	12.86
Warwick, 02887	12.86
Warwick, 02888	12.86
Warwick, 02889	12.86
West Greenwich, 02817	7.22
West Kingston, 02892	4.74
West Warwick, 02893	12.86
Westerly, 02891	4.74
Wood River Junction, 02894	4.74
Woonsocket, 02895	11.18
Wyoming, 02898	4.74

**Figure 2:**
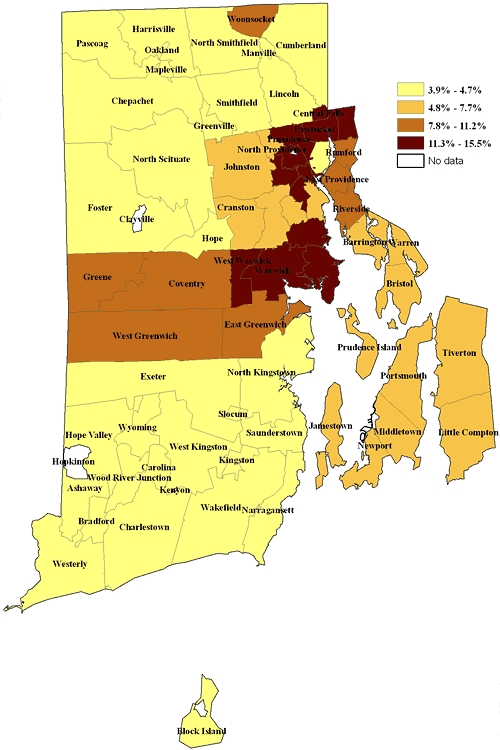
Prevalence of current depression, by Rhode Island cities and towns by zip code, data from the Depression and Anxiety module (Patient Health Questionnaire 8) of the 2006 Rhode Island Behavioral Risk Factor Surveillance System.

The prevalence of current depression in Providence (excluding the affluent east side), Central Falls, Pawtucket, Warwick, and West Warwick ranged from 11.3% to 15.5% (Figure 2). The first 3 cities are urban with a high proportion of low-income and minority residents. The prevalence in Woonsocket, East Providence, Coventry, Greene, West Greenwich, and East Greenwich ranged from 7.8% to 11.2%. Other than East Providence, these are suburban areas. The remainder of the state, with the lowest rates for current depression, is largely suburban and rural.

**Table:** 

**Town or City, Zip Code**	**Current Depression, %**
Adamsville, 02801	6.62
Albion, 02802	3.93
Ashaway, 02804	4.71
Barrington, 02806	6.61
Block Island, 02807	4.71
Bradford, 02808	4.71
Bristol, 02809	6.61
Carolina, 02812	4.71
Central Falls, 02863	12.87
Charlestown, 02813	4.71
Chepachet, 02814	3.93
Coventry, 02816	9.57
Cranston, 02910	7.72
Cranston, 02920	7.72
Cranston, 02921	7.72
Cumberland, 02864	3.93
East Greenwich, 02818	9.57
East Providence, 02914	8.42
Exeter, 02822	4.71
Fiskeville, 02823	7.72
Forestdale, 02824	3.93
Foster, 02825	3.93
Glendale, 02826	3.93
Greene, 02827	9.57
Greenville, 02828	3.93
Harmony, 02829	3.93
Harrisville, 02830	3.93
Hope Valley, 02832	4.71
Hope, 02831	3.93
Jamestown, 02835	6.62
Johnston, 02919	7.02
Kingston, 02881	4.71
Lincoln, 02865	3.93
Little Compton, 02837	6.62
Manville, 02838	3.93
Mapleville, 02839	3.93
Middletown, 02842	5.74
Narragansett, 02882	4.71
Newport, 02840	5.74
North Kingstown, 02852	4.71
North Kingstown, 02854	4.71
North Providence, 02911	7.02
North Scituate, 02857	3.93
North Smithfield, 02896	3.93
Oakland, 02858	3.93
Pascoag, 02859	3.93
Pawtucket, 02860	12.87
Pawtucket, 02861	12.87
Pawtucket, 02862	12.87
Portsmouth, 02871	6.62
Providence, 02901	12.39
Providence, 02902	12.39
Providence, 02903	12.39
Providence, 02904	12.39
Providence, 02905	7.72
Providence, 02906	3.90
Providence, 02907	12.39
Providence, 02908	12.39
Providence, 02909	12.39
Providence, 02912	12.39
Prudence Island, 02872	6.61
Riverside, 02915	8.42
Rockville, 02873	4.71
Rumford, 02916	8.42
Saunderstown, 02874	4.71
Slatersville, 02876	3.93
Slocum, 02877	4.71
Smithfield, 02917	3.93
Tiverton, 02878	6.62
Wakefield, 02879	4.71
Wakefield, 02880	4.71
Warren, 02885	6.61
Warwick, 02886	15.49
Warwick, 02887	15.49
Warwick, 02888	15.49
Warwick, 02889	15.49
West Greenwich, 02817	9.57
West Kingston, 02892	4.71
West Warwick, 02893	15.49
Westerly, 02891	4.71
Wood River Junction, 02894	4.71
Woonsocket, 02895	11.21
Wyoming, 02898	4.71

## Discussion

Our analysis showed that any of the 3 shorter items provide results comparable with those of the PHQ-8 in estimating overall prevalence of depression and mental distress, identifying high-risk populations, and identifying significant associations with risk variables. We recommend use of any of the 3 shorter items as a proxy on the BRFSS or similar population-based surveys to obtain a population estimate of depression prevalence. Any one of the 3 is adequate for use in descriptive analyses of population data. They provide an efficient means of assessing depression prevalence in adult populations when survey efficiency precludes use of the longer PHQ-8. However, the PHQ-8 should be preferred for surveys requiring a high level of sensitivity as well as specificity, accuracy in reliably assigning depression severity status to specific respondents, or requiring assessment over time of population changes in depression severity.

The strengths of the PHQ-8 are its high degree of both sensitivity (88%) and specificity (88%) for major depression (6,9), its adequacy for diagnosis, its ability to assess severity of depression, and its validation in the general population (6,9,10). The weakness of the PHQ-8 is its length, which makes it time-consuming to administer and answer, and its complex scoring algorithm. Although the simple question measures of depression are inadequate for diagnosis (9), they are useful for screening for depression. Sad/blue/depressed and frequent mental distress are single questions, very easy to answer and administer, with few training requirements. The PHQ-2 is easy to answer and administer, and the scoring algorithm is simple (9).

The test characteristics and the test performance of the PHQ-2 were more sensitive than the 2 HRQOL indicators. Frequent mental distress was more concordant with the PHQ-8 than was the PHQ-2 in distinguishing demographic groups and risk factors with higher prevalence of depression. Based on the AUC, sad/blue/depressed is better than frequent mental distress and the PHQ-2. The proportion of records with missing values was lower for the 2 single HRQOL items than for the PHQ-2 or the PHQ-8. No measure was better than the others in all respects.

Both the sad/blue/depressed and the PHQ-8 maps showed that the prevalence of depression was highest in the core urban areas of the state and lowest in the more suburban and rural areas. The differences between the 2 distributions may reflect the greater power of the PHQ-8 to discriminate between cities and towns. This discriminatory power may also be reflected in the wider range of values resulting from the PHQ-8 measure than from the sad/blue/depressed measure.

Rhode Island is a small state, so the study population is homogeneous compared with populations of other states. Our analyses went beyond simple comparisons of test characteristics and test performance to include distinguishing levels of demographic characteristics and risk factors as well as within-state geographic comparisons.

Some limitations of this analysis should be noted. First, the HRQOL indicators are based on a 30-day recall period, while the PHQ-2 and PHQ-8 are based on a 14-day recall period. We have no way to assess the effect of this difference in recall periods. Second, both sets of questions (the HRQOL and the PHQ-8) were asked in the same interview session (ie, they were not context-independent). We have no way to assess the effect of this. Finally, because the calculation of PHQ-8 and PHQ-2 scores require responses to all questions used in calculating scores, it was necessary to impute missing values. In the future, we need to vary the cut points of the 2 single-question screeners and the PHQ-2 to optimize their performance against the PHQ-8 (30).

We conclude that, for Rhode Island, any of the 3 short screeners is sufficiently specific and sensitive to provide population prevalence estimates of depression and can be used for descriptive analyses of our population survey data. Mapping of these depression estimates also indicates localities where the need for mental health services is greatest. To validate the generalizability of our findings, it will be important to replicate them in other states' BRFSS surveys.

## Figures and Tables

**Table 1:** Comparison of Test Characteristics and Test Performance of 3 Indicators Against PHQ-8, Rhode Island, 2006^a^,^b^

Measures	HRQOL		D&A Module

	Frequent Mental Distress, % (95% CI)	Sad/Blue/Depressed, % (95% CI)	PHQ-2, % (95% CI)
Sensitivity	59.4 (51.9-66.9)	59.7 (52.2-67.3)	66.8 (59.4-74.1)
Specificity	94.4 (93.3-95.6)	96.4 (95.7-97.2)	96.1 (95.1-97.0)
Positive predictive value	50.0 (42.7-57.3)	60.7 (53.6-67.7)	61.4 (54.0-68.8)
Negative predictive value	96.1 (95.3-97.0)	96.3 (95.4-97.1)	96.9 (96.0-97.7)

Abbreviations: PHQ, patient health questionnaire; HRQOL, health-related quality of life; D&A, depression and anxiety; CI, confidence interval.

a Data are reported as weighted percentages.

b PHQ-8 was the standard used for comparison.

**Table 2:** Percentage of Depression and Mental Distress Indicators for Selected Demographic Characteristics and Risk Factors (n = 4,515), Rhode Island Adults, 2006^a^

Demographic Characteristics and Risk Factors	No.^b^ (%)	HRQOL		D&A Module	

		Frequent Mental Distress^c^ (n = 438), % (95% CI)	Sad/Blue/ Depressed^c^ (n = 434), % (95% CI)	Current Depression (PHQ-2) (n = 404), % (95% CI)	Current Depression (PHQ-8) (n = 339), % (95% CI)
**Age, y**		*P* = .003	*P* = .37	*P* = .07	*P* = .001
18-44	1,485 (49.6)	11.5 (9.2-13.8)	8.4 (6.4-10.4)	11.0 (8.7-13.4)	9.7 (7.5-11.9)
45-64	1,719 (32.2)	10.7 (9.0-12.3)	10.0 (8.5-11.6)	9.2 (7.7-10.8)	9.2 (7.6-10.7)
≥65	1,255 (18.3)	6.5 (4.9-8.0)	8.8 (7.0-10.6)	7.8 (6.0-9.7)	4.5 (3.0-5.9)
**Sex**		*P* = .01	*P* = .002	*P* = .05	*P* < .001
Men	1,652 (47.5)	8.4 (6.5-10.4)	6.9 (5.3-8.6)	8.4 (6.3-10.4)	6.2 (4.5-7.9)
Women	2,863 (52.5)	11.9 (10.2-13.7)	10.8 (9.2-12.3)	11.1 (9.4-12.8)	10.7 (8.9-12.4)
**Annual income, $**		*P* < .001	*P* < .001	*P* < .001	*P* < .001
<25,000	954 (19.3)	21.6 (17.5-25.8)	24.1 (19.8-28.4)	23.0 (18.8-27.2)	21.9 (17.4-26.4)
25,000-49,999	996 (25.1)	10.5 (7.5-13.5)	8.4 (6.3-10.5)	11.4 (8.4-14.4)	8.6 (6.3-10.8)
≥50,000	1,843 (55.6)	6.2 (4.8-7.7)	3.9 (2.7-5.0)	4.6 (3.1-6.0)	4.4 (3.1-5.7)
**Employment status**		*P* < .001	*P* < .001	*P* < .001	*P* < .001
Employed	2,581 (64.0)	7.7 (6.3-9.0)	5.4 (4.4-6.5)	6.8 (5.5-8.2)	6.1 (4.9-7.3)
Retired	1,121 (16.5)	6.2 (4.5-7.8)	6.6 (5.0-8.2)	7.4 (5.6-9.2)	4.3 (2.9-5.7)
Homemaker/student	362 (10.4)	13.9 (7.8-20.0)	12.8 (7.7-18.0)	9.6 (4.3-14.9)	7.0 (3.0-10.9)
Unemployed	171 (4.8)	21.8 (11.4-32.3)	20.6 (10.4-30.8)	21.4 (10.5-32.2)	25.8 (13.3-38.3)
Unable to work	261 (4.4)	43.5 (35.7-51.2)	47.9 (40.2-55.7)	50.9 (42.8-59.1)	50.4 (41.8-59.0)
**Current smoker**		*P* < .001	*P* < .001	*P* < .001	*P* < .001
No	3,731 (80.8)	8.1 (6.8-9.3)	7.2 (6.1-8.4)	7.6 (6.4-8.9)	6.3 (5.1-7.4)
Yes	762 (19.2)	19.8 (15.8-23.9)	16.3 (12.6-19.9)	19.1 (14.9-23.2)	18.4 (14.1-22.6)
**Chronic drinker**		*P* = .15	*P* = .08	*P* = .93	*P* = .49
No	4,174 (94.0)	10.0 (8.7-11.3)	8.7 (7.6-9.8)	9.9 (8.5-11.2)	8.4 (7.2-9.7)
Yes	226 (6.0)	15.2 (7.0-23.5)	14.8 (6.4-23.2)	9.5 (2.9-16.2)	10.9 (3.3-18.4)
**Leisure-time physical activity**		*P* < .001	*P* < .001	*P* < .001	*P* < .001
Yes	3,282 (75.3)	8.4 (6.9-9.9)	6.0 (4.9-7.2)	7.3 (6.0-8.7)	6.1 (4.8-7.3)
No	1,227 (24.7)	16.1 (13.3-18.9)	17.9 (15.0-20.7)	17.5 (14.4-20.6)	16.5 (13.3-19.8)
**Asthma**		*P* < .001	*P* < .001	*P* < .001	*P* < .001
No	4,008 (89.5)	9.1 (7.8-10.3)	8.2 (7.0-9.3)	8.8 (7.5-10.1)	7.1 (6.0-8.2)
Yes	479 (10.5)	21.1 (15.5-26.8)	15.8 (10.9-20.6)	18.9 (13.4-24.4)	22.1 (15.6-28.6)
**Diabetes**		*P* = .02	*P* < .001	*P* < .001	*P* < .001
No	4,091 (92.6)	10.0 (8.6-11.3)	8.4 (7.2-9.6)	9.3 (7.9-10.6)	8.0 (6.7-9.3)
Yes	421 (7.4)	14.5 (10.3-18.7)	15.8 (11.6-19.9)	16.6 (12.0-21.2)	15.5 (10.9-20.2)
**Obesity^d^**		*P* < .001	*P* = .003	*P* = .04	*P* < .001
No	3,281 (78.6)	9.2 (7.7-10.7)	8.1 (6.7-9.4)	9.3 (7.7-10.9)	7.1 (5.7-8.5)
Yes	973 (21.4)	15.0 (12.1-17.9)	12.0 (9.6-14.4)	12.3 (9.7-14.9)	14.6 (11.3-17.9)
**Disability**		*P* < .001	*P* < .001	*P* < .001	*P* < .001
No	3,357 (80.2)	6.9 (5.6-8.2)	4.8 (3.8-5.8)	6.0 (4.7-7.2)	4.3 (3.2-5.4)
Yes	1,073 (19.8)	19.5 (16.0-23.1)	21.4 (17.8-25.0)	21.5 (17.8-25.2)	21.7 (17.8-25.6)

Abbreviations: HRQOL, health-related quality of life; D&A, depression and anxiety; CI, confidence interval; PHQ, patient health questionnaire.

a Data are reported as weighted percentages. *P* values calculated by using the *χ* ^2^ test.

b Numbers may not equal total because of missing data.

c Reported this indicator for ≥14 days/month. See Methods section for complete variable description.

d Obesity was defined as body mass index >30 kg/m^2^.

**Table 3:** Depression and Mental Distress Indicators, by Demographic Characteristics and Risk Factors, Rhode Island Adults, 2006^a^

Demographic Characteristics and Risk Factors	HRQOL		D&A Module	

	Frequent Mental Distress,^b^ AOR (95% CI)	Sad/Blue/Depressed,^b^ AOR (95% CI)	Current Depression (PHQ-2), AOR (95% CI)	Current Depression (PHQ-8), AOR (95% CI)
**Age, y**				
18-44	2.3 (1.5-3.6)	1.2 (0.8-2.0)	2.4 (1.5-3.9)	3.9 (2.2-6.8)
45-64	1.9 (1.3-2.8)	1.3 (0.8-1.9)	1.5 (1.0-2.4)	2.8 (1.7-4.6)
≥65	1 [Reference]	1 [Reference]	1 [Reference]	1 [Reference]
**Sex**				
Men	1 [Reference]	1 [Reference]	1 [Reference]	1 [Reference]
Women	1.3 (0.9-1.8)	1.4 (1.0-2.0)	1.2 (0.9-1.7)	1.7 (1.2-2.5)
**Annual income, $**				
<25,000	2.6 (1.8-3.8)	4.0 (2.6-6.3)	3.4 (2.2-5.4)	3.4 (2.1-5.5)
25,000-49,999	1.4 (0.9-2.2)	1.8 (1.2-2.7)	2.0 (1.3-3.2)	1.6 (1.0-2.6)
≥50,000	1 [Reference]	1 [Reference]	1 [Reference]	1 [Reference]
**Employment status**				
Employed	1 [Reference]	1 [Reference]	1 [Reference]	1 [Reference]
Retired	0.9 (0.6-1.4)	0.6 (0.4-1.0)	1.0 (0.7-1.6)	0.9 (0.6-1.6)
Homemaker/student	1.7 (1.0-3.0)	1.8 (1.1-3.0)	1.1 (0.5-2.1)	1.0 (0.5-1.8)
Unemployed	1.8 (0.9-3.5)	2.0 (0.9-4.2)	1.7 (0.8-3.6)	2.1 (1.0-4.7)
Unable to work	2.7 (1.7-4.4)	3.0 (1.9-4.9)	3.4 (2.2-5.5)	3.1 (1.8-5.1)
**Current smoker**				
No	1 [Reference]	1 [Reference]	1 [Reference]	1 [Reference]
Yes	2.1 (1.5-3.0)	1.8 (1.2-2.5)	2.0 (1.4-2.9)	2.3 (1.6-3.2)
**Chronic drinker**				
No	1 [Reference]	1 [Reference]	1 [Reference]	1 [Reference]
Yes	1.9 (0.9-3.7)	2.6 (1.3-5.3)	1.1 (0.5-2.5)	1.6 (0.7-3.4)
**Leisure-time physical activity**				
Yes	1 [Reference]	1 [Reference]	1 [Reference]	1 [Reference]
No	1.2 (0.9-1.7)	1.8 (1.3-2.5)	1.5 (1.0-2.1)	1.7 (1.2-2.4)
**Asthma**				
No	1 [Reference]	1 [Reference]	1 [Reference]	1 [Reference]
Yes	1.6 (1.0-2.4)	1.0 (0.7-1.7)	1.3 (0.8-1.9)	1.7 (1.0-2.7)
**Diabetes**				
No	1 [Reference]	1 [Reference]	1 [Reference]	1 [Reference]
Yes	1.0 (0.7-1.6)	1.2 (0.8-1.8)	1.2 (0.8-1.8)	1.1 (0.7-1.8)
**Obesity^c^**				
No	1 [Reference]	1 [Reference]	1 [Reference]	1 [Reference]
Yes	1.4 (1.0-2.0)	1.1 (0.8-1.6)	1.0 (0.7-1.4)	1.5 (1.0-2.2)
**Disability**				
No	1 [Reference]	1 [Reference]	1 [Reference]	1 [Reference]
Yes	2.5 (1.8-3.7)	3.5 (2.5-5.0)	2.9 (2.1-4.1)	4.5 (3.0-6.7)

Abbreviations: HRQOL, health-related quality of life; D&A, depression and anxiety; AOR, adjusted odds ratio; CI, confidence interval; PHQ, patient health questionnaire.

a Data are reported as AORs by all other variables in the model after multiple imputation to account for missing data in survey samples (26,27). AOR is considered significant if its confidence interval does not include 1.

b Defined as reporting this indicator for 14 or more days per month. See Methods section for complete variable description.

c Obesity was defined as body mass index >30 kg/m^2^.
